# Wave-Net: A Marine Raft Aquaculture Area Extraction Framework Based on Feature Aggregation and Feature Dispersion for Synthetic Aperture Radar Images

**DOI:** 10.3390/s25072207

**Published:** 2025-03-31

**Authors:** Chengyi Wang, Lei Wang, Ningyang Li

**Affiliations:** 1 Aerospace Information Research Institute, Chinese Academy of Sciences, Beijing 100094, China; wanglei98@aircas.ac.cn; 2 Key Laboratory of Earth Observation of Hainan Province, Hainan Aerospace Information Research Institute, Sanya 572029, China; ningyang799@gmail.com

**Keywords:** raft aquaculture area, semantic segmentation, SAR, feature aggregation, feature dispersion, deep learning

## Abstract

Monitoring raft aquaculture areas plays an important role in the sustainability of marine aquaculture. With the advantages of full-time observation and ability to penetrate clouds, synthetic aperture radar (SAR) imaging has replaced laborious on-site investigation and has become the preferred approach. However, the existing deep learning-based semantic segmentation approaches generally suffer from speckle noise and have difficulty with multi-scale structures, which blurs the boundaries of raft aquaculture areas, and therefore, they connect them incorrectly. To cope with this problem, a wave-shaped neural network (Wave-Net), which is mainly composed of a feature aggregation part and a feature dispersion part, was proposed. Its feature aggregation part extracts both global and local features from different scales of raft aquaculture areas with asymmetric V-shaped subnetworks. Then, its feature dispersion part uses asymmetric Ʌ-shaped subnetworks to refine the boundaries of different scales of raft aquaculture areas. During these processes, both residual connections and reconstruction losses are adopted between the identical scales of feature maps to promote feature fusion and parameter optimization. The experimental results revealed that the proposed Wave-Net model solved the issue of blurred boundaries and achieved better segmentation accuracy with limited samples.

## 1. Introduction

In the past two decades, the marine aquaculture industry, especially raft aquaculture [1,2], has developed rapidly to meet the growing demand for marine food. Raft aquaculture mainly contains of floating rafts and cages [3]. However, some disordered and dense raft aquaculture areas caused great pressure on the ocean environment, which is not good for sustainability. To relieve this issue, it is necessary to monitor raft aquaculture areas in a timely fashion through effective methods. Because raft aquaculture areas are generally widely distributed and far from shore, on-site investigations are laborious. Remote sensing technology can observe large areas efficiently and periodically. Considering the interference of clouds and fog with information acquisition, this work chose synthetic aperture radar (SAR) images to monitor marine raft aquaculture areas.

As a coherent imaging system, there are a number of noises caused by the coherent superposition of waves emitted by randomly distributed scatterers, such as speckle noise, during the imaging procedure of SAR. Thus, the scattering characteristics [4] of objects will be deviated in SAR images, which often blur the boundaries of raft aquaculture areas. At the same time, the multi-scale spatial distribution of raft aquaculture areas is affected by the environmental factors, including noises and waves. These weaken the global features and blur the local features, which makes it difficult to extract the raft aquaculture areas accurately from SAR images.

Recently, many deep learning-based methods have been proposed for computer vision tasks, including classification [5,6], object detection [7,8], and segmentation [9,10,11]. In particular, these methods significantly improve the image extraction accuracy of raft aquaculture areas. The popular methods include convolutional neural networks [12,13], regional convolutional neural networks [14], fully convolutional networks [15], U-Net [16,17], DeepLab [18,19], Transformer [20,21], etc. However, there are still some challenges in extracting raft aquaculture areas accurately, i.e., the single information of SAR images and the complex interference factors. To handle these challenges, deeper neural networks with more parameters should be constructed and a large number of samples are needed to avoid the issues of overfitting and disappearing gradients. In this way, it is possible for these neural networks to possess stronger generalization ability and better segmentation performance.

Current research on the extraction of raft aquaculture areas from SAR images can be divided into two categories. One is to improve the quality and quantity of samples. For example, Liu et al. [17] obtained more samples with flip and mirror operations. Zou et al. [22] adopted sample enhancement and converted multiple bands into a pseudo color image. Deng et al. [23] overlaid samples and supplemented negative samples to enhance their sample library. However, these methods are laborious and generally find it difficult to achieve efficient extraction. Another approach to improving the extraction accuracy is by optimizing the feature representation ability of the model. For example, Zhang et al. [24] proposed a TransUNet model that combined channel attention [25], multi-scale asymmetric convolution, and shaping of prior knowledge constraints to optimize a U-Net backbone network, which achieved higher extraction accuracy. A data fusion strategy was also applied to consider the information from different types of images [26,27], which takes into full account the advantages of different sensors. Although these methods introduced special modules to enhance specific features, there were some uncertain factors during the training procedures, thereby suppressing the representation ability of these networks.

In summary, both inherent coherent speckle noises in SAR images and complex background interference cause models to fail to extract finely the boundaries of raft aquaculture areas. Based on the residual connection and encoder-decoder architecture, this article proposed a wave-shaped network (Wave-Net) model that cascaded a feature aggregation part and a feature dispersion part. Compared with other methods, the proposed end-to-end Wave-Net model not only reduces the complexity and number of parameters, but also enhances the feature representation ability, which avoids laborious consumption to build a sample library. Experimental results on limited samples showed that the Wave-Net model achieved higher segmentation accuracy and recognition accuracy.

The main contributions of this article are as follows:

(1) A feature aggregation part, which contains multiple asymmetric V-shaped subnetworks, was designed to represent the global and local features. It accurately extracts the boundaries of raft aquaculture areas.
(2) A feature dispersion part, which contains multiple asymmetric Ʌ-shaped subnetworks, was designed to refine the boundaries of the raft aquaculture areas. The number of Ʌ-shaped subnetworks is the same as the number of V-shaped subnetworks used to construct the encoder-decoder architecture, which makes full use of the multi-scale information of raft aquaculture areas.

The rest of this article is arranged as follows. First, Section 2 details both the data and the proposed Wave-Net model. Then, Section 3 reports the experimental results. Next, discussions about the feasibility and limitations of the proposal are presented in Section 4. Finally, this article is concluded in Section 5.

## 2. Data and Methods

### 2.1. Research Regions

**Figure 1:**
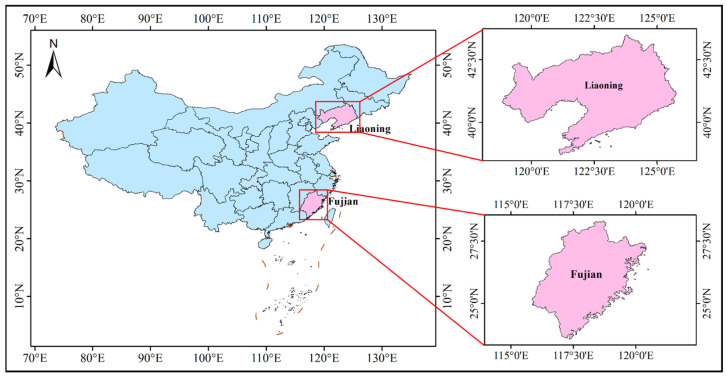
Research regions for raft aquaculture areas extraction.

As shown in Figure 1, the marine raft aquaculture areas in Liaoning and Fujian provinces, China were selected as the research regions. Liaoning province is located in the northeast of China and is the northernmost coastal province in China. The southern Liaodong Peninsula extends into the Yellow Sea and Bohai Sea and is surrounded by the sea on three sides. There are rich fishery resources and many islands in the marine regions of Liaodong Peninsula. It contains 266 islands, a coastline of 2292.4 km, and an island coastline of 627.6 km. Fujian province is located on the southeast coast of China and adjacent to the East China Sea and the South China Sea. The mainland coastline is 3752 km long, which ranks first in China. The tortuosity of the coastline also ranks first. The number of bays comprises one-sixth of the national bays. The natural conditions for developing marine industries, especially for raft aquaculture, are superior.

### 2.2. Data

**Figure 2:**
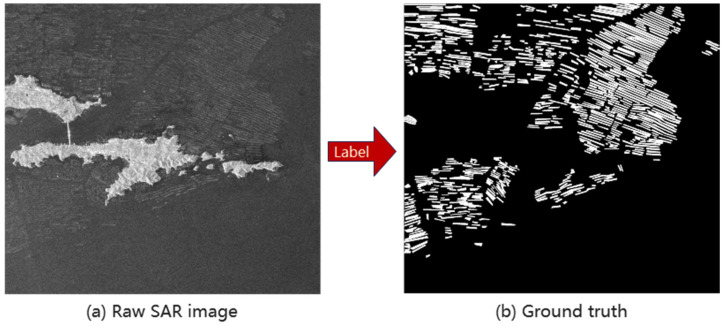
SAR image and corresponding ground truth map.

In this article, two recent SAR images captured on 18 May 2021 (Liaoning) and 14 March 2021 (Fujian) were acquired on the Sentinel-1 Science Data Center website (https://search.asf.alaska.edu/ (accessed on 24 October 2024). Both are VV polarized Level-1 Ground Range Detected (GRD) Sentinel-1. Figure 2a shows both images preprocessed on the Sentinel Application Platform (SNAP), including radiometric calibration, speckle noise removal using the 7 × 7 Refined Lee filter, and terrain correction using the SRTM1 Sec HGT data.

Marine raft aquaculture areas in SAR images were marked manually by expert interpreters using ArcGIS tools and saved as the ground truth map shown in Figure 2b. Then, both the SAR image and ground truth map were cropped into 685 blocks with a size of 320 × 320. Each block will be a sample for training and testing. The number of samples was expanded from 685 to 4110 by horizontal flipping, vertical flipping, diagonal mirroring, etc. All samples were divided into a training set, validation set, and test set at a ratio of 3:1:1.

### 2.3. Wave-Net Model

#### 2.3.1. Overview of Wave-Net Model

**Figure 3:**
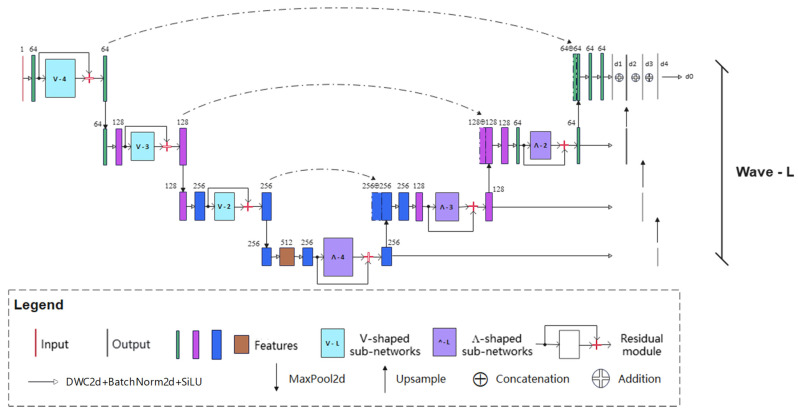
Architecture of the Wave-Net model.

As shown in Figure 3, the proposed end-to-end Wave-Net model is composed of a feature aggregation part (left), a feature dispersion part (right), and a multi-scale loss fusion part (end). It is a semantic segmentation framework combining V-shaped and Ʌ-shaped subnetworks, encoder–decoder architecture, and residual connections, which look like waves. Specifically, the kernel sizes of all depth-wise convolutional (DWC) layers in the Wave-Net model are set to 3 × 3 to capture the local features of raft aquaculture areas. The batch normalization layer and SiLU activation function are followed to regulate the feature distribution non-linearly. To prevent overfitting and disappearing gradients caused by network depth, residual connections were deployed in each subnetwork of the two parts. The identical levels of features in the feature aggregation and dispersion parts were fused to take into consideration different scales of feature details.

SAR images were cropped into many blocks as the samples because the network depth and model size depend on the size of the sample to some degree. Samples with a larger size have a wider image view and relatively smaller raft aquaculture area. A deeper network should be constructed to capture the complete texture information. At the same time, a large number of samples are required for optimization. On the contrary, the boundaries of raft aquaculture areas generally cannot be represented in samples with smaller sizes, which is adverse to the fine extraction of boundary information. In this work, limited samples (less than 1000) were used for training the proposed Wave-Net model. According to the sample size, there are three architectures (W-L and L = 3, 4, and 5) for the proposed model, as presented in Table 1.

#### 2.3.2. Feature Aggregation Part

**Figure 4:**
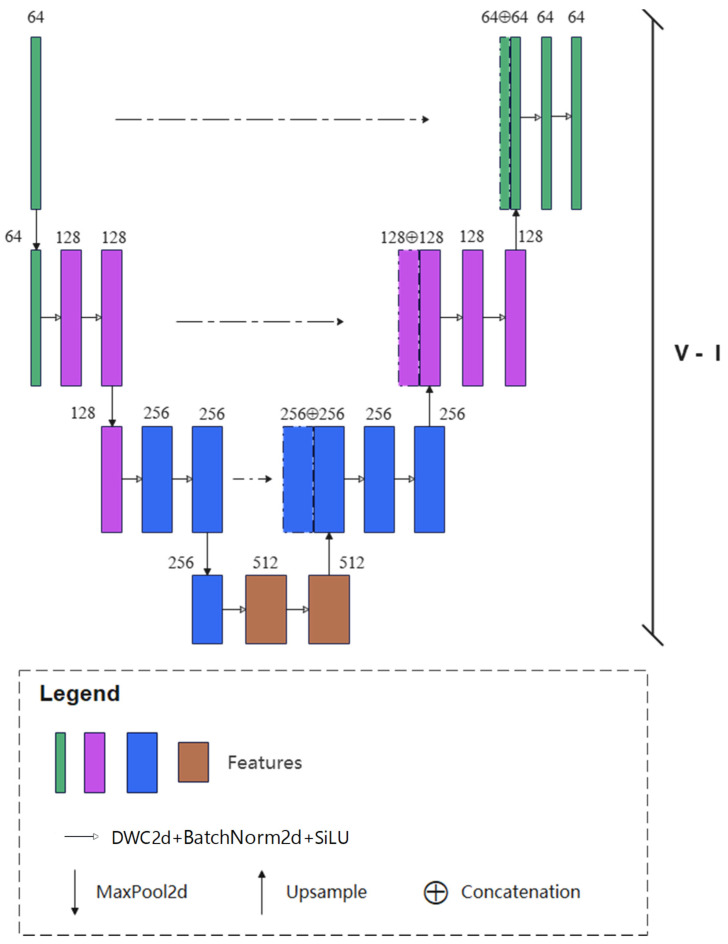
First V-shaped subnetwork of the feature aggregation part, where dashed line denotes the identical map.

To capture the contextual information of different scales of raft aquaculture areas in SAR images and reduce computational complexity, the feature aggregation part was designed to extract both global and local features. This part contains multiple asymmetric V-shaped sub-networks and the number of sub-networks varies with the sample size. As shown in Figure 4, different scales of features can be extracted through a series of two-dimensional convolutional layers. In the stage of encoding, the max-pooling layers are used to retain the salient information and reduce the sizes of feature maps. Then, the sizes of feature maps will be restored through the bilinear up-sampling layers so that the identical levels of feature maps can be fused with the corresponding scale of features in the previous encoding stage. The output of the current V-shaped subnetwork is encoded by the pooling operation and will be the input of the subsequent V-shaped subnetwork. The depth of each V-shaped subnetwork in the feature aggregation part decreases gradually to extract fully the multi-scale features of the raft aquaculture area in the SAR image.

#### 2.3.3. Feature Dispersion Part

**Figure 5:**
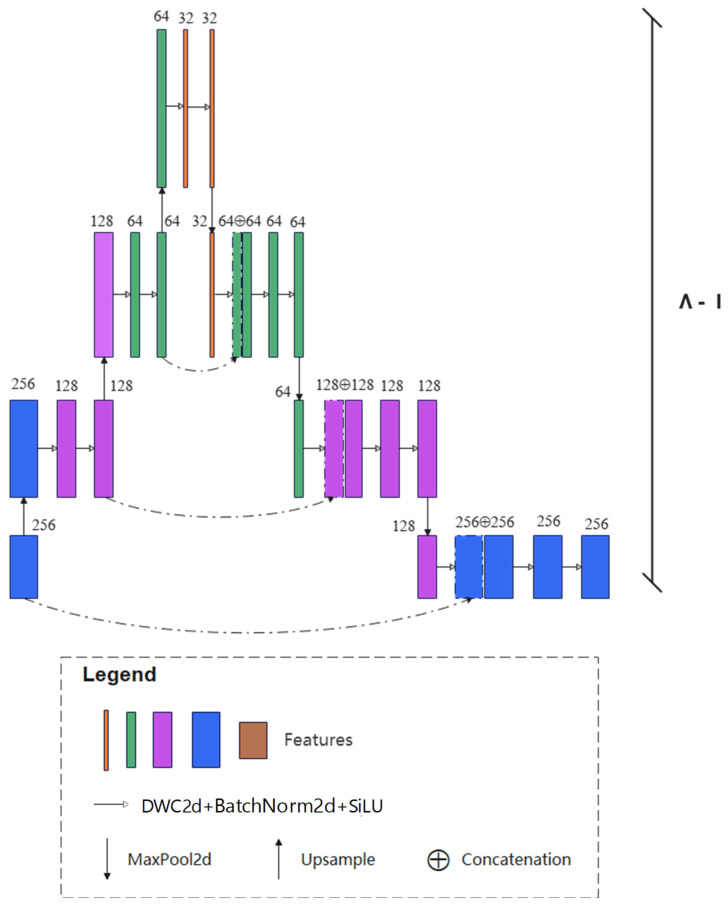
First Λ-shaped subnetwork of the feature dispersion part, where dashed line denotes the identical map.

The feature dispersion part aims to resolve two issues. One is the information loss caused by the up-sampling operations during feature decoding. Another is that the networks focus excessively on the shallow features and neglect the deep and abstract features during feature encoding, which generally blurs the boundaries of the raft aquaculture areas. The feature dispersion part contains multiple asymmetric Λ-shaped subnetworks. As shown in Figure 5, the dimensions of the feature maps are first increased via up-sampling layers and then reduced with convolutional layers. Details of the boundaries of the raft aquaculture areas will be refined during this process. The number of Λ-shaped subnetworks is the same as that of the V-shaped subnetworks. Similarly, the output of each Λ-shaped subnetwork is decoded by an up-sampling operation and sent to the next subnetwork. The depth of each Λ-shaped subnetwork decreases gradually to recover the feature details of raft aquaculture areas.

#### 2.3.4. Multi-Scale Loss Fusion Part

Loss function is used to measure the difference between prediction and true label. It makes the parameters update during back propagation possible. In the proposed Wave-Net model, the output of each level of V-shaped subnetwork is fused the output of the corresponding level of Λ-shaped subnetwork for measuring the binary cross entropy (BCE) loss. As shown in Figure 3, four BCE losses are used to optimize the parameters of the whole model.

## 3. Experiments

### 3.1. Implementation

During the training process, the number of iterations and batch size were set to 200 and four, separately. An Adam optimizer was adopted to update the parameters of the model. The (learning rate, decay factor) were set to (0.00001, 0.001). All experiments were conducted on a workstation with an NVIDIA GeForce RTX 4080 GPU, NVIDIA, Santa Clara, CA, USA. The deep learning framework is PyTorch 1.3.

**Figure 6:**
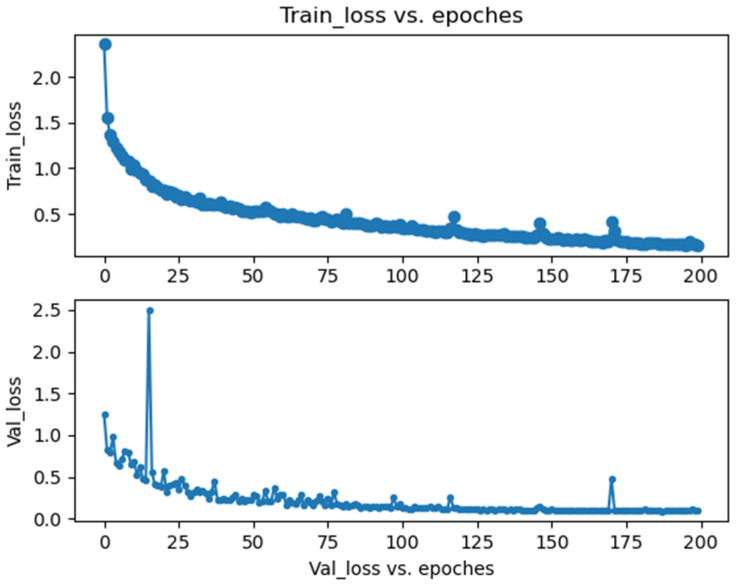
Visualization of training loss and validation loss.

Figure 6 shows the curves of the training and validation losses. It can be seen that both kinds of loss trend downward smoothly. There is no obvious difference between the two curves in terms of the trend. The validation loss began to converge after about 150 epochs, which reveals that the training data have been exploited fully for promoting the performances of the model. At the end, the loss curves converged to below 0.2, which achieved the relatively ideal optimization of the proposed model.

### 3.2. Quantitative Evaluation

To evaluate the segmentation performances of different segmentation methods, including DeepLabV3 [28], U-Net [29], and U^2^-Net [30], some quantitative metrics, including precision, recall, F1 score, and interaction of units (IoU), were selected. All experiments were repeated five times to relieve the influence of random parameters. The average and standard deviations of these metrics on two data sets are reported in Table 2 and Table 3. The bold values are the best in corresponding columns.

It can be seen from Table 2 that the precision of the U-Net model is higher than that of the DeepLabV3 model. The F1 scores and IoUs of both models are almost the same while the recall of the U-Net model is slightly lower than that of the DeepLabV3 model. This is because there is generally an inverse relationship between recall and precision. For the improvement of the U-Net model, four metrics of the U^2^-Net model are higher those of the U-Net model on both data sets. From Table 2 and Table 3, the U^2^-Net model obtained the best segmentation performance among the three methods. However, four metrics, except the recall on Liaoning data set, of the proposed Wave-Net model are better than those of the other models. Therefore, the overall performance of the Wave-Net model is superior.

### 3.3. Qualitative Evaluation

**Figure 7:**
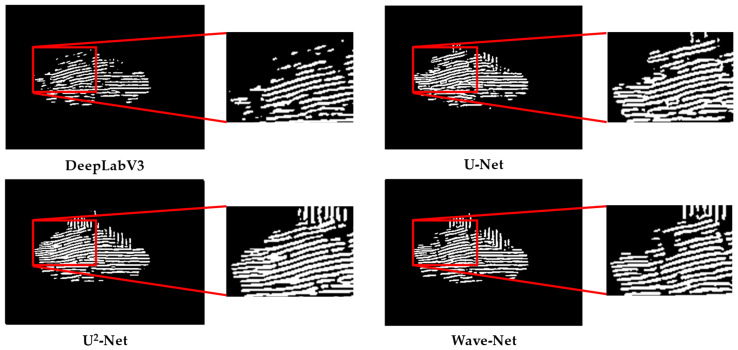
Visualization of the segmentation results of different methods in the Shandong scenario.

**Figure 8:**
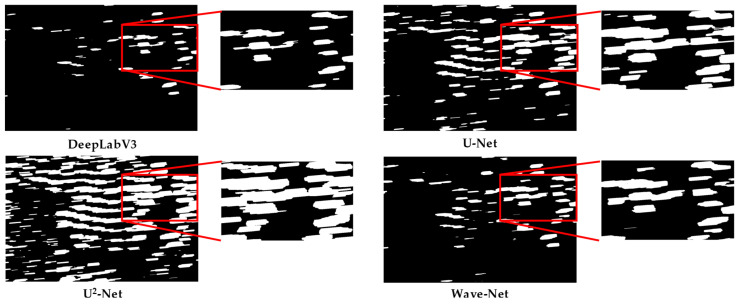
Visualization of the segmentation results of different methods in the Fujian-1 scenario.

**Figure 9:**
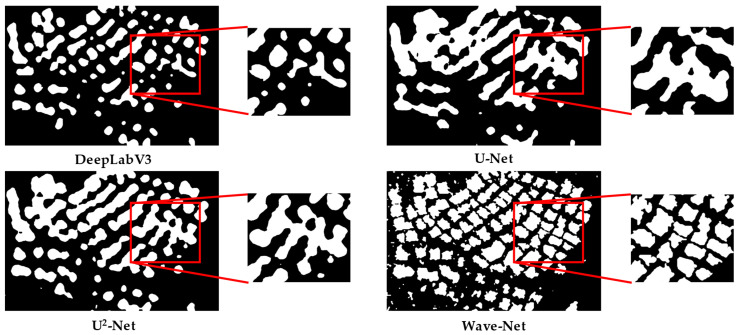
Visualization of the segmentation results of different methods in the Fujian-2 scenario.

**Figure 10:**
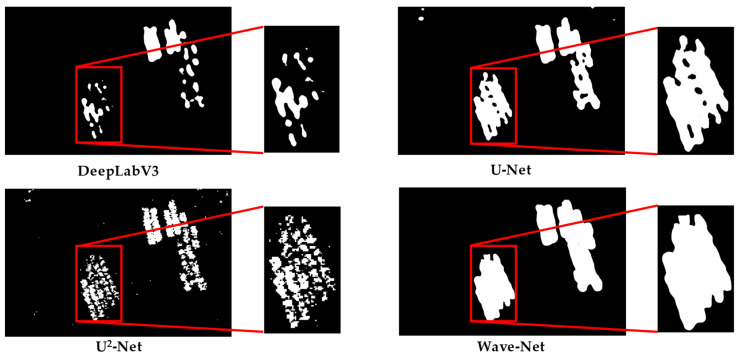
Visualization of the segmentation results of different methods in the Fujian-3 scenario.

The segmentation maps of different methods in the Shandong, Fujian-1, Fujian-2, and Fujian-3 scenarios are presented in Figure 7, Figure 8, Figure 9 and Figure 10 for qualitative evaluation. The original SAR images were cropped to many blocks with a size of 320 × 320 for prediction. It can be observed from these figures that the segmentation results of DeepLabV3 are the worst because there are many incomplete and blurring raft aquaculture areas. Compared to DeepLabV3, the other two methods, U-Net and U^2^-Net, improved the raft aquaculture areas, but there are some wrong connections between different raft aquaculture areas that do not exist in real scenarios. On the contrary, the Wave-Net model not only achieved more complete prediction areas than the other models, but also clarified the boundaries of the raft aquaculture areas (amplified in red rectangles).

### 3.4. Ablation Study

Residual connections can relieve the issue of the vanishing gradient for the deep models. The proposed Wave-Net model deploys the residual connections inside the V-shaped subnetworks and Ʌ-shaped subnetworks to promote the optimization of different levels of features. To verify the rationality of this setting, those residual connections were removed for segmentation. As shown in Table 4, the performances of the models without residual connections all declined, especially on the F1 metric. By comparing the final losses of the two schemes, it can be concluded that the model without residual connections needs to be optimized further. This also demonstrates the effectiveness of residual connections.

## 4. Discussion

### 4.1. Feasibility Analysis

The speckle noise of SAR images generally can weaken the boundaries of raft aquaculture areas. Multi-scale spatial structures of raft aquaculture areas also make it difficult for models to represent the global and local features. Hence, these factors suppress the segmentation performances of the deep learning-based models. This article proposed a Wave-Net model that contains feature aggregation and dispersion parts to suppress speckle noise and highlight the boundaries of raft aquaculture areas. Inside both parts, the V-shaped and Λ-shaped sub-networks were cascaded to fully extract the multi-scale global and local features. The experimental results show that the Wave-Net model achieves better segmentation performances and eliminates the wrong connections between raft aquaculture areas (shown in Figure 7, Figure 8, Figure 9 and Figure 10).

Among the three methods for comparison, although the DeepLabV3 model introduced dilated convolution to reduced number of parameters, its ability to represent the multi-scale features is limited. In addition, it neglected the effects of speckle noise, which lost many aquaculture areas and caused unclear boundaries. The U-Net model combined a symmetrical encoder–decoder structure and a residual mechanism to preserve more details than the DeepLabV3 model. The U^2^-Net model increased the network depth by nesting the U-shaped structure and fused the segmentation result of each layer. However, it still cannot distinguish accurately the boundaries of some aquaculture areas. In summary, the segmentation performance of the Wave-Net model is better than the other models.

### 4.2. Limitations

Quality samples play a vital role in semantic segmentation. Although the proposed Wave-Net model has achieved superior segmentation performances, its accuracy in prediction scenarios decreased to some extent compared to that of the test experiment. The possible reasons are as follows:

(1) Raft aquaculture areas in different regions contain different textures and features. The current training set captured in the Liaoning and Fujian datasets does not contain the marine raft aquaculture areas in Shandong. Therefore, the generalization ability of the Wave-Net model decreased slightly when extracting marine raft aquaculture areas in other regions.
(2) There are severe random speckle noises in SAR images at different times. Meanwhile, the gray-scale values of the same marine raft aquaculture area vary greatly at different times. Hence, some deviations may occur when using neural networks for segmenting raft aquaculture areas. Therefore, it is necessary to develop more effective algorithms to further reduce speckle noises and generate more samples to enhance the robustness of the model.

## 5. Conclusions

This article proposed a Wave-Net model for extracting marine raft aquaculture areas from SAR images. Specifically, a feature aggregation part, which contains multiple asymmetric V-shaped subnetworks, first captured the multi-scale global and local features of raft aquaculture areas. Then, a feature dispersion part distinguished clearly the boundaries of raft aquaculture areas and suppressed the speckle noise with an identical number of Ʌ-shaped subnetworks. A residual connection was applied to promote the optimization of parameters and relieve the overfitting. Experimental results on limited samples demonstrated the structural rationality of the Wave-Net model and its superior segmentation performance. The prediction results in a real scenario showed that the Wave-Net model achieved satisfactory extraction results.

## 6. Patents

The novel marine raft aquaculture extraction method employed in this study is based on the principles outlined in China Patent No [2023110740726], field [24 August 2023], inventors [Yanjun Guo, Chengyi Wang, Bin Yu, Guo Yu, Yunyan Du, Wenping Li, and Chengyong Wu].

## Figures and Tables

**Table 1:** Different sample sizes and corresponding network architecture.

Sample size (pixel × pixel)	[300 × 300, 400 × 400]	[400 × 400, 500 × 500]	[500 × 500, 600 × 600]
Network architecture	L = 3	L = 4	L = 5

**Table 2:** Segmentation results of different methods on the Liaoning data set.

Models	Precision (%)	Recall (%)	F1 (%)	IoU (%)
DeepLabV3 [15]	78.95 ± 1.96	**72.96** ± 2.23	80.91 ± 2.05	67.98 ± 2.84
U-Net [16]	80.75 ± 1.17	71.20 ± 1.48	80.97 ± 1.28	68.04 ± 1.82
U^2^-Net [17]	87.19 ± 1.89	71.89 ± 2.07	82.86 ± 2.16	68.75 ± 1.83
**Wave-Net**	**89.54** ± 1.47	70.81 ± 2.67	**83.46** ± 1.68	**69.85** ± 1.52

**Table 3:** Segmentation results of different methods on the Fujian data set.

Models	Precision (%)	Recall (%)	F1 (%)	IoU (%)
DeepLabV3 [15]	80.32 ± 1.67	71.23 ± 2.11	81.15 ± 1.81	65.61 ± 1.75
U-Net [16]	82.21 ± 2.11	72.33 ± 1.65	82.33 ± 1.95	67.81 ± 1.91
U^2^-Net [17]	86.33 ± 1.65	75.55 ± 2.12	85.23 ± 1.33	71.51 ± 1.38
**Wave-Net**	**89.10** ± 1.32	**81.21** ± 1.46	**90.12** ± 1.35	**75.51** ± 1.55

**Table 4:** Segmentation results of the proposed model with and without the residual connections.

Residual	Precision (%)	Recall (%)	F1 (%)	IoU (%)	Final Loss (Train/val)
No	85.04	68.85	76.55	65.62	0.1389/0.1246
Yes	87.56	70.44	80.09	67.12	0.1233/0.1137

## Data Availability

Data are contained within the article.
